# Clinical Wound Healing After Lower Third Molar Surgery with Envelope and Bayonet Flaps: A Randomized Clinical Trial

**DOI:** 10.3390/mps8050101

**Published:** 2025-09-04

**Authors:** Roberto Pippi, Chiara Mazzei, Alessandra Pietrantoni

**Affiliations:** Department of Odontostomatological and Maxillo Facial Sciences, Sapienza University of Rome, 00185 Roma, RM, Italy; roberto.pippi@uniroma1.it (R.P.); chiara.mazzei@uniroma1.it (C.M.)

**Keywords:** third molar extraction, wound healing, flap design

## Abstract

Objectives: The present study mainly aimed to identify whether the envelope and triangular flaps affected wound healing and patient quality of life differently. Secondarily, the study aimed to investigate whether some anatomical and operational variables may also affect healing. Study design: A prospective randomized study was conducted with 56 fully impacted lower third molars, randomly divided into two groups, one treated with the envelope flap and the other with the bayonet flap. Qualitative variables were transformed into quantitative ones and then analyzed using independent samples *t*-tests or analysis of variance. An analysis of bivariate correlations with Pearson’s coefficient was also used. The chi-square test was used to verify the association between each flap and the categorical variables considered. Results: No statistically significant associations were found between flap types and dehiscence, although the mean dehiscence diameter was consistently greater in the envelope flap group. The maximum diameter of the dehiscence at 14 days was found to be significantly and negatively related to the 14-day wound healing indices. Analyses relating to the quality of life did not show significant associations. Conclusions: Despite some significant healing differences between the two considered flaps exist, they do not have relevant effects on the patient’s post-operative quality of life.

## 1. Introduction

Lower third molar (LTM) surgery is a routine practice, often associated with complications such as pain, swelling, trismus and post-operative infections [1]. The impact of these complications on the patient’s daily routine, although temporary, is an aspect of great clinical relevance. In fact, several studies found variations in patient quality of life (QOL) after surgical removal of LTMs [2,3], but only a few studies [4] investigated whether QOL differs in relation to the performed flap. Other studies attempted to evaluate whether the incidence of post-extraction complications varied depending on the type of surgical flaps [5]. Among LTM surgery complications, dehiscence has not been frequently assessed, although it is one of the most common complications. Dehiscence is characterized by complete or partial diastasis of the edges of the sutured surgical wound due to early suture loosening/breakage or marginal tissue laceration [6]. Following LTM surgery, it especially occurs distal to the second molar and can cause the accumulation of food and debris in the extraction site. This can not only contribute to the development of halitosis but also promote the onset of infection, thus delaying healing of the surgical wound and causing discomfort and pain to patients.

Given the importance of access flap design and management, some authors have already examined these aspects in relation to dehiscence associated with the extraction of fully impacted LTMs [7].

Both local and systemic factors are considered to predispose flap dehiscence occurrence.

Local factors involve the following: edema; trauma to the wound resulting from chewing and/or oral hygiene at home; sutures placed too close to the incision line, with increased risk of tearing; intramural tensions; flap repositioning with the incision line just above the residual bone cavity; ischemia, characterized by decreased oxygen and nutrient supply; flap lacerations, due to compromised integrity of the periosteum and consequent significant reduction in osteogenic potential; thick suture threads or traumatic flap management [6]; wide diameter of the post-extraction socket with insufficient filling by the blood clot; phlogogenic suture materials, such as silk [8], which predispose to bacterial proliferation; and inadequate tension at the surgical wound margins [9]. Excessive tension can lead to flap ischemia, which is also exacerbated by post-operative edema, while insufficient tension slows the healing process.

Systemic factors that influence the healing process of surgical wounds include all those conditions that can hinder neo-angiogenesis, immune response and rate of collagen production, especially diabetes [10], hypovitaminosis [11] and glucocorticoid-based therapies [12]. Lastly, tobacco significantly reduces perfusion at the healing site, promoting vasoconstriction and platelet aggregation, and compromises the body’s immune response, reducing the effectiveness of neutrophils in fighting infections [13].

Since dehiscence is related to prolonged healing times and increased post-operative patient discomfort, due to food accumulation, pain and increased risk of infections [3], it can be considered a clinical expression of an abnormal healing process. Given the paucity of literature on the incidence of dehiscence, the type of healing and the impact on the quality of life of patients undergoing lower third molar extraction in relation to the type of surgical flap, the main aim of the present study was to identify whether the two most common flaps for LTM surgery, that is the envelope flap and the triangular flap with an intra-sulcular incision, may differently affect wound healing, assuming the dehiscence as a sign of bad wound healing. The secondary aim was to investigate whether some anatomical and operational variables may affect healing after LTM extraction. Finally, the present study also aimed to investigate whether a disparity in the patient’s QOL was present after surgery, in relation to the two types of flaps.

## 2. Materials and Methods

### 2.1. Patient Selection

Fifty-six patients were included. The sample size calculation was based on the effect size reported by Sandhu et al. [14]. Using this effect size, a sample of 28 LTMs, extracted for each type of flap, would have provided 95% power to detect an odds ratio of 0.0977 and to reject the null hypothesis that the two groups could have the same proportion of post-surgical dehiscence, using a two-tailed binomial Fisher’s exact test.

All surgeries were performed at the Oral Surgery Complex Unit of the Head and Neck Integrated Care Department, within the dental division of Umberto I University hospital, Rome, by post-graduate students in Oral Surgery and tutors.

### 2.2. Inclusion Criteria

Adult patients of all races and both genders, in whom a completely impacted LTM was planned for extraction, were included.

### 2.3. Exclusion Criteria

Patients undergoing pharmacological treatment for systemic diseases capable of influencing the healing process, pregnant women, smokers, disabled individuals and all surgeries in which intra-operative complications involving soft tissues occurred would been excluded. Such complications included incision irregularities, lacerations, perforations, abrasions and crushing, with evident deformation of the flap.

Surgeries were randomly divided into two groups: in the first group, surgeries performed with an envelope flap were included, while in the second group, surgeries performed with an intrasulcular bayonet flap were included. Each surgery was assigned an identification number for randomization, which was carried out via an Excel sheet, in which random numbers that identified surgeries to be included in the same group were collected. Surgeries whose identification number was present on the Excel sheet were assigned to the first group, while the others were assigned to the second group.

When extraction was planned, patients signed informed consent forms to participate in the study and to allow the use of their personal data for statistical purposes.

According to the most recent Cochrane review [15] about the prevention of infections following tooth extraction, available when the study was planned, all patients underwent antibiotic prophylaxis with 2 g of amoxicillin plus clavulanic acid, 1 h before surgery.

**Figure 1:**
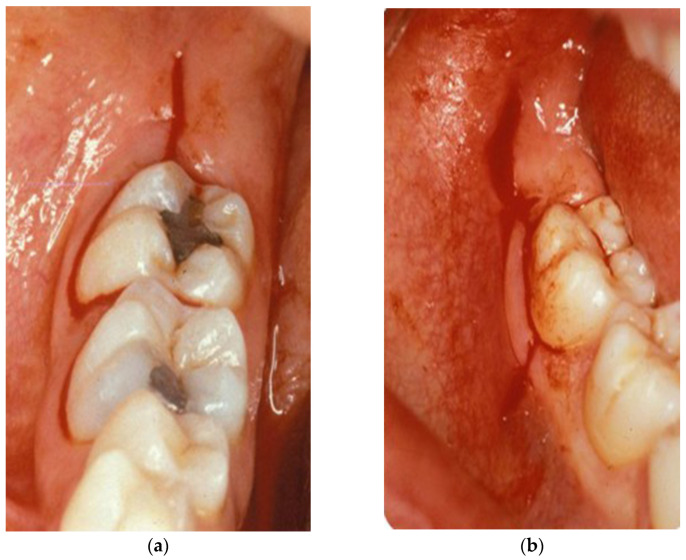
Envelope (**a**) and bayonet (**b**) flaps.

Extractions were performed under local anesthesia and according to a standard protocol that applied the same technique and the same suture material (4-0 silk) to both groups, except for flap design. The envelope flap was used in the first group and the intrasulcular bayonet flap in the second group. The envelope flap, or marginal flap, involved two incision lines: the first intrasulcular incision started from the mesio-buccal angle of the first molar and ended at the disto-buccal angle of the second molar; the second incision started from the distal end of the first line and was disto-buccally directed toward the ascendent rhamus (Figure 1a). The intrasulcular bayonet flap involved three incision lines: the first started from the mesio-buccal angle of the second molar and extended downwards, obliquely and forwards; the second started from the coronal end of the first line and extended intra-sulcularly along the second molar, until reaching the most distal portion of the latter; the third ran from the distal end of the previous line, directed disto-buccally toward the ascendent rhamus (Figure 1b).

The study was approved by the institutional Ethical Committee with the approval number 5478, and it was also registered on the ClinicalTrials.gov platform with the ID number NCT04314765. The guidelines on ethical principles for medical research involving human subjects, as provided by the World Medical Association’s Declaration of Helsinki (October 2013) were followed.

### 2.4. Data Collection

The following clinical pre-operative data were recorded: patient gender, age and race, pre-operative symptoms (yes, no), inter-incisor height, Full Mouth Plaque Score (FMPS) and the Plaque Score (PS) of the involved quadrant, probing depth distal to the second molar, amount of keratinized gingiva and the position of the gingival margin relative to the second molar cemento-enamel junction (CEJ).

The following radiographic data were recorded: site of impaction, type of impaction (osteo-mucosal/completely osseous), Pell and Gregory class (1–3), depth of impaction, tooth position (mesio-angular, disto-angular, horizontal, vertical), root number (1, 2, more than 2) and morphology (fused/separated not divergent, divergent), relationships with the second molar (contiguous, superimposed). As for the depth of impaction, the Wilson classification was used as already modified by Pippi et al. [16], dividing the C class in two subclasses, C1 and C2.

The following clinical intra- and post-operative data were also recorded: the maximum diameter of the residual bone cavity, the maximum depth of the residual bone cavity in relation to the second molar CEJ, the position of the gingival margin after suturing, compared to the second molar CEJ and the duration of surgery.

The patients in both groups underwent examination 2 days after surgery, on the seventh post-operative day, when the sutures were removed, and 14 days after surgery. On all these occasions, the following data were recorded: the clinical wound healing index (WHI), the maximum dimensions of any possible dehiscence, the PS of the involved quadrant, the inter-incisive height, any possible post-operative complications and any post-operative pharmacological treatment.

During the clinical follow-up, post-operative edema was also evaluated and classified as mild, moderate or severe.

The WHI was specifically created by modifying the Wound Evaluation Scale (WES) [17], as previously applied by other authors [18], thus yielding a score ranging from 0 to 9. It assessed the following 9 parameters, each with a dichotomous score: bleeding (present = 0, absent = 1); suppuration (present = 0, absent = 1); swelling (present = 0, absent = 1); stepped edges (present = 0, absent = 1); contour irregularities/wrinkling (present = 0, absent = 1); wound edge separation greater than 2 mm (present = 0, absent = 1); inversion of the edges (present = 0, absent = 1); gingival color (totally/partially red = 0, pink = 1); overall cosmetic appearance (not good = 0, good = 1). A total score of 9 indicates optimal wound healing, while lower scores indicate suboptimal wound healing.

**Figure 2:**
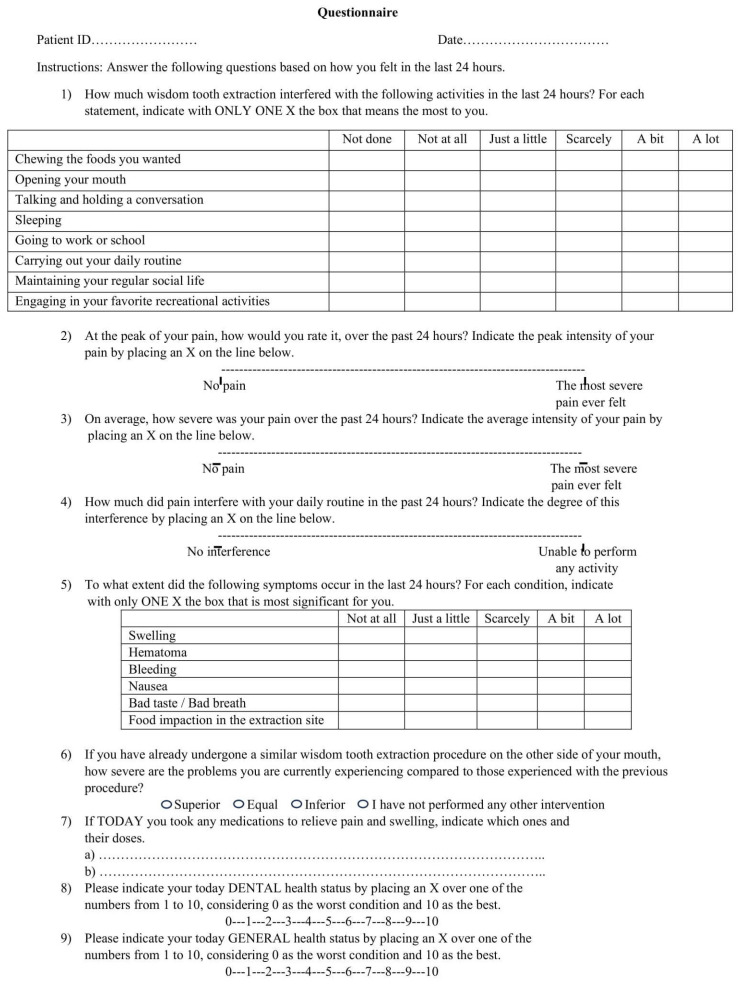
Health-Related Quality of Life Questionnaire (modified based on Shugars et al. [19]).

The Health-Related Quality of Life (HRQOL) questionnaire, appropriately modified by Shugars et al. [19] for studies similar to the present one (Figure 2), was translated into Italian and administered to all patients pre-operatively, as well as 2, 5, 7 and 14 days after surgery. It addressed 4 different areas: oral functions, daily activities, signs and symptoms associated with post-operative complications and pain.

Oral function was investigated using three questions about the ability to chew foods, open the mouth, talk and hold a conversation. The ability to carry out daily activities was investigated using 5 questions about sleeping, going to work or school, carrying out daily routine, maintaining social life and engaging in recreational activities. Unlike the study by Shugars [19], for each of the 8 questions above, the patients were asked to mark an “x” next to one of the following answers, without knowing the score assigned: not done (0), not at all (1), just a little (2), scarcely (3), a bit (4), a lot (5). As for pain, three questions investigated its maximum and average intensity and how it interfered with the daily activities using three different bipolar visual analogue scales (VASs) which, unlike the Shugars questionnaire [19], were typically represented by a straight line with only one of the two opposite conditions on each end, “not at all” and “the most severe condition never felt”, without intermediate level definitions or scoring levels. The following six other post-operative symptoms were investigated: swelling, hematoma, bleeding, nausea, bad taste/bad breath and food impaction in the extraction site. For each of them, patients had to put an “x” sign on one of the following answers which, unlike the study by Shugars et al. [19], did not have the assigned score visible: not at all (1), just a little (2), scarcely (3), a bit (4) and a lot (5). Another question asked the patients to compare the severity of symptoms to those experienced during any previous similar oral surgery. Possible answers were “superior”, “equal”, “inferior” and “not undergone to other similar surgeries”. This question replaced the original item in the questionnaire that asked which extraction caused the most problems. Patients were also asked if they were taking any medication on the day of the assessment. Lastly, for both dental and general health status, a 0–10 numerical scale was administered to the patients.

Post-operative clinical data were recorded at 2, 5 and 7 days after surgery, with all recordings performed by the same surgeon, who was not involved in the study and who was unaware of the nature of the study.

### 2.5. Statistical Analysis

SAS Local (X64_10PRO) was used for statistical analysis. Frequency and percentage analyses were carried out to describe all categorical variables. Descriptive analyses, including the calculation of both the mean and the standard deviation, were used for the quantitative variables. The transformation of some qualitative variables into quantitative ones was carried out to verify the existence of statistically significant associations between the type of flap performed, post-extraction healing index and patient QOL. These variables were then analyzed using independent samples *t*-tests, when comparing only two groups, or alternatively, through analysis of variance (ANOVA) when more than two groups were involved. The analysis of bivariate correlations with the Pearson correlation coefficient was also used to study the quantitative variables. The same associations were examined in depth using the Kaplan–Maier survival curves. To this end, maximum, minimum and average values of each quantitative variable were used. Finally, the chi-square test was used to verify the existence of significant associations between the type of flap and the categorical variables considered. Results with a *p*-value <0.05 were considered significant.

## 3. Results

### 3.1. Descriptive Analysis

All descriptive data are summarized in Table 1. Surgeries were performed on 56 patients with an average age of 23.7 ± 4.48 years. Fifty-five of them were Caucasian, 38 were females (67.86%) and 18 males (32.14%). In only 16.07% of the sample, pre-operative symptoms were present. Third molars were almost equally distributed on the left (51.79%) and right sides (48.21%). Most of them (50 = 89.29%) had two roots, which were mostly separate and non-divergent (38 = 76%). The majority also presented with osteo-mucosal impaction (48 = 85.7%), were classified as Pell and Gregory Class 2 (44 = 78.6%) and showed mesio-angular (29 = 51.79%) and horizontal (16 = 28.57%) positions and a depth of impaction classified as B/C1 (32 = 57.14%; 15 = 26.79%). A radiographic overlap with the second molar was present in only 14.3% of cases.

As regards the pre-operative periodontal examination, the average probing value distal to the second molar was 5 ± 2.1 mm, and the average amount of attached gingiva in the same region was 2.5 ± 0.81 mm. A coronal position of the soft tissues compared to the CEJ of the second molar was found in 76.8% of cases, pre-operatively, and it did not change immediately after surgery. No intra-operative accidents involving the soft tissues occurred in any of the surgeries.

As for the intra-operative data, the maximum diameter of the residual bony cavity was on average 12 mm, and its maximum depth, compared to the second molar CEJ was on average 2.5 mm. Finally, the average durations were, on average, 1520 ± 878.83 s for surgery and 48 ± 288.85 s for suturing.

### 3.2. Inferential Analysis

Four different sub-objectives were investigated:

1. To assess differences in healing between the two types of flaps using the wound dehiscence as a principal parameter of healing. The null hypothesis was that the two groups could have the same proportion of post-surgical dehiscence.
2. To evaluate whether the possible presence of dehiscence had any repercussions on wound healing. For each test, the null hypothesis was that there was no influence on healing.
3. To investigate whether a correlation existed between clinical, anatomical and operational variables and surgical wound healing. For each test, the null hypothesis was that no influence on healing was associated with each variable.
4. To find possible associations between the flap design and any changes in terms of post-surgical patient’s QOL. The null hypothesis was that there would be no differences in terms of QOL between one kind of flap and the other.

**Figure 3:**
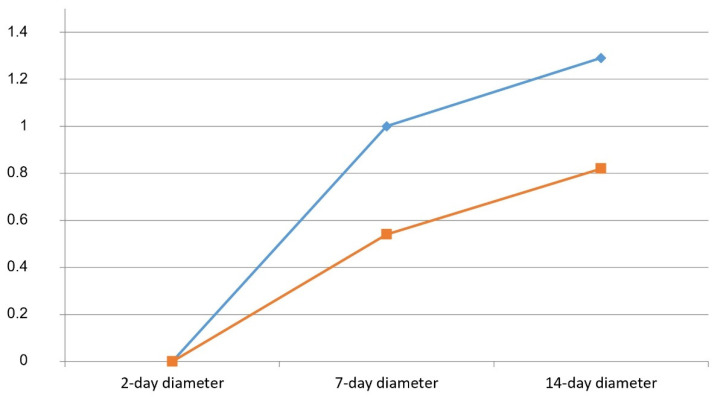
Time course of dehiscence by flap type.

Regarding the first objective, no statistically significant associations were found between the type of flap (envelope or intra-sulcular bayonet) and the presence of dehiscence. In addition, considering the maximum diameter of the dehiscence, the independent samples *t*-test showed no statistically significant differences between the surgeries performed with the envelope flap and those performed with the bayonet flap (14-day follow-up: *t* = 1.045; *p*= 0.300; 7-day follow-up: *t* = 0.976; *p* = 0.333), although the mean diameter of the dehiscence in the cases with the envelope flap was always greater (average diameter at 7 days: 1.00 mm; average diameter at 14 days: 1.26 mm) than that in those with the intra-sulcular bayonet flap (average diameter at 7 days: 0.54 mm; average diameter at 14 days: 0.83 mm—Table 2, Figure 3).

Regarding the second proposed objective, no significant correlation was found between the presence of dehiscence and post-surgical healing.

For this investigation, an analysis of variance was carried out, verifying whether the type of flap and the possible presence of dehiscence could influence the WHI, measured at 2, 7 and 14 days. in addition, a new healing index variable (general healing index, GHI), was introduced for statistical purposes, calculated as the difference between the WHI values detected at 14 and 2 days, respectively. Regarding the GHI, the results showed that the presence of the dehiscence and not the type of flap (F_(1,52)_ = 5.21; *p* = 0.7023) influenced healing (Table 3), as the interaction effect lost significance when considering the type of flap. Specifically, when dehiscence was absent, the GHI showed higher scores (M = 1.85; sd = 1.90) compared to when dehiscence was present (M = 0.65; sd = 1.85). Although the interaction effect was not significant, the data showed that the lowest GHI scores were found in the cases treated with the envelope flap (M = 0.17; sd = 1.80—Table 4).

The chi-square test was used to verify whether significant associations were present between dehiscence and edema at 2, 7 and 14 days after surgery. The results showed an association approaching significance between severe edema and the presence of dehiscence 2 days after surgery (χ^2^ = 4.92; *p* = 0.085). In particular, 66.7% of patients with dehiscence presented severe edema. No significant associations were found between edema and dehiscence at 7 and 14 days after surgery.

The associations between maximum diameter of dehiscence at 7 and 14 days and the WHI at 2, 7 and 14 days, including the GHI, were measured through a correlation analysis carried out with the Pearson’s r coefficient. According to the data, the maximum diameter of dehiscence at 14 days was found significantly and negatively related to the 14-day WHI (r = −0.493; *p* < 0.001) and the GHI (r = −0.325; *p* = 0.015—Table 5).

As for the third aim, no difference was found between males and females in terms of healing at 2, 7 and 14 postoperative days, although males tended to report higher values. Furthermore, age was not associated with healing at 2, 7, and 14 postoperative days. Among anatomical variables, statistically significant results were found only for the variable “root morphology”. In particular, the chi-square test showed a significant association between the presence of dehiscence at 2 days and LTMs with apical root anomalies (χ^2^= 18.63; *p* < 0.001). Furthermore, the analysis of variance found a significant association between root morphology and both the GHI (F_(3,52)_ = 3.076; *p* = 0.036) and the 14-day WHI (F_(3,52)_ = 2.814; *p* = 0.048). In particular, post-hoc comparisons highlighted that LTMs with divergent roots had higher healing rates than those with apical root anomalies. As regards the operational variables, the correlation analysis highlighted a negative correlation between the residual cavity depth and the 7-day WHI (r = −0.317; *p* = 0.017). No significant correlations were found between the residual cavity depth and both the WHI, measured at 2 and 14 days, and the GHI. Furthermore, the maximum diameter of the residual cavity and the durations of both surgery and suturing were not associated with any of the healing indices.

Analyses relating to the fourth objective did not show significant associations. However, a trend toward statistical significance was observed for the type of flap in relation to the average pain measured 2 days after surgery (*t* = 1.761; *p* = 0.084) and the swelling detected at 14 days (*t* = −1.947; *p* = 0.059). Specifically, 2 days after surgery, the average intensity of pain was higher in the patients treated with the envelope flap (M = 3.86; sd = 2.43) compared to those who had been treated with the bayonet flap (M = 2.75; sd = 2.27). Differently, 14 days after surgery, the swelling was more frequently reported by patients treated with the bayonet flap (M = 1.43; ds = 0.69) compared to those in whom the envelope flap was used (M = 1.14; ds = 0.36). No other associations between QOL and type of flap were found.

## 4. Discussion

### 4.1. Flap Design and Dehiscence

The results obtained from the present study showed that the incidence of dehiscence was comparable in the envelope flap and the bayonet flap. This disagrees with some studies who found the dehiscence significantly more frequently in the envelope flap than in the triangular one [4,7,20], although the recent literature review by Zhu et al. [21] did not find any statistical difference.

On the contrary, considering the maximum diameter of the dehiscence, differences were found in relation to flap type. In particular, the average diameter of the dehiscence in cases treated with the envelope flap was always greater (average diameter at 7 days: 1.00 mm; average diameter at 14 days: 1.26 mm) compared to the cases treated with the intra-sulcular bayonet flap (average diameter at 7 days: 0.54 mm; average diameter at 14 days: 0.83 mm). The average diameter of the dehiscence, at the 7-day control visit, reached double values in cases treated with an envelope flap, and this difference remained almost unchanged 14 days after surgery. This finding was also reported by Rahpeyma et al. [20], in whose study 67% of cases treated with the envelope flap showed a dehiscence exceeding 5 mm in diameter after 7 days post-extraction, compared to 19% of cases treated with the triangular flap.

### 4.2. Effects of Dehiscence on Healing

Unlike previous studies [22,23], the present study did not find significant association between the presence of dehiscence and healing. In fact, poorer healing observed in patients who developed a dehiscence after being treated with an envelope flap is associated with more post-operative complications, such as edema, pain and trismus [21,24,25]. However, the present data showed that the maximum diameter of the dehiscence at 14 days was significantly and negatively related to the 14-day WHI (r = −0.493; *p* < 0.001) and the GHI (r = −0.325; *p* = 0.015—Table 5). Moreover, although the present results do not reach statistical significance, the patients who presented larger average dehiscence diameters, namely those who were treated with the envelope flap, exhibited poorer healing compared to the patients with smaller dehiscence diameters, who were treated with the intra-sulcular bayonet.

In fact, in the presence of an early wide dehiscence, the healing process can be compared to that of secondary intention. In this regard, a recent review [26] found only slight differences between primary and secondary closure techniques in third molar surgery. However, the review did not assess the early post-operative wound healing, but rather focused on symptoms and signs associated with healing, including pain, swelling, trismus, infection and bleeding.

In the present study, an association approaching statistical significance was also found for edema (χ^2^ = 4.92; *p* = 0.085), which is another common parameter of healing, since 66.7% of patients with dehiscence presented severe edema (Figure 3).

In this regard, it is possible that severe edema involving the flap is responsible for the tissue yielding at the point where the suture thread passes. As the edema subsides, the resulting decrease in tension of the suture thread at that site can cause dehiscence [27].

### 4.3. Influence of Anatomical and Operative Variables on Healing

Early healing after LTM surgery has not been extensively investigated in relation to all clinical, anatomical and operational variables. Age and sex have been investigated in other kinds of oral surgery for a possible association with better or worse wound healing [28] but not specifically in LTM surgery. Although oral mucosal wounds were found to heal more slowly in males and in older patients [29], in the present study, no significant differences were found, consistent with the findings of Drumond et al. in gingivectomy [28], despite the difference in mean patient age (23.7 ± 4.48 vs 26.08 ± 6.7 years) between the two samples.

In the present study, a statistically significant correlation was found between healing and root morphology of the extracted LTM: as morphological complexity increased, healing outcomes worsened. In particular, apical anomalies were significantly associated with the presence of dehiscence during the 2-day follow-up (χ^2^ = 18.63; *p* < 0.001). Similar results were also observed for the GHI (F_(3,52)_ = 3.076; *p* = 0.036) and the WHI at 14 days (F_(3,52)_ = 2.814; *p* = 0.048). Healing was also poorer in cases of apical anomalies than in cases of divergent roots. Furthermore, a significant negative correlation was found between the residual cavity depth and the 7-day WHI (r = −0.317; *p* = 0.017). Although the literature does not provide information in this regard, these results highlighted that the complex root morphology of the LTM may have a significant impact on the surgical procedure and the patient’s subsequent recovery. A possible explanation of this result can be that root morphology contributes to increasing surgery complexity, thus requesting more invasive and longstanding procedures, which may increase the trauma exerted on the flap, such as stretching and compression, with consequent ischemia and tissue suffering [30]. In fact, the average duration of surgeries on LTMs with complex root anatomy such as highly divergent roots (1890 s) or roots with apical anomalies (1620 s) was consistently longer than the average duration of all surgeries (1520 s), although these data are not shown in tables.

Likewise, a wide depth of the residual cavity, although essential in certain clinical situations, significantly influences healing [25,31,32]. In particular, a deep residual cavity is generally associated with more invasive surgery, which may correlate with a higher risk of complications, including infections, bleeding and damage of the surrounding tissue. Extensive removal of bone tissue can also alter the structural stability of the treated area, especially in areas exposed to functional load. This can negatively affect proper formation and maintenance of the blood clot, which is essential for the healing process [33]. Insufficient clotting can also increase the risk of alveolar osteitis [34,35].

Therefore, to reduce the risk of bad healing, it can be suggested that in cases of root anomalies and deep impaction, surgeries should be performed by more experienced surgeons in order to reduce the operating times and the trauma exerted on the flaps. Proper postoperative monitoring may also help to reduce the impact of the above factors. During the first two weeks after oral surgery, the flap’s adherence to the underlying hard tissues is guaranteed only by the blood clot, and even though the wound edges are sutured, mobility, whether spontaneous or related to function, will still be present. This may interfere with the stabilization of the clot, leading to bleeding and delaying wound healing. For this reason, it is advisable to establish a follow-up protocol to detect any signs and symptoms related to surgical complications affecting the flap and manage them appropriately, ensuring optimal healing [6]. In the presence of root anomalies, and when a wide ostectomy is expected, patients should also be preventively informed about a possible poorer post-operative course to ensure a truly informed consent to surgery.

The fact that, among all anatomical and operational variables considered, only root morphology and residual cavity depth were found to be statistically related with healing may be due to the small size of the sample, with a consequent low number of cases for each variable analyzed. The relationship between healing and anatomical and operational variables has not been extensively investigated in literature. The study by Phillips et al. [36], although found that age, sex, prior symptoms related to the third molars and the surgeon’s perception of difficulty were statistically significant predictors of delayed clinical healing, also failed to find a statistical relationship between healing and both LTM position and surgical time.

No significant relationships were also found, in the pertinent literature, between both anatomical and operational variables and adjacent second molar periodontal healing after LTM surgery [37,38].

### 4.4. Influence of Flap Type on QOL

Patient perception of health-related quality of life (HRQOL) is nowadays considered to be as important as the common clinical and radiographic treatment outcome measures. Since the HRQOL questionnaire proposed by Shugars et al. [18] expressly evaluates the short-term outcomes of third molar surgery, it was used in the present study.

Unlike the study of Şimşek Kaya et al. [4], the type of flap did not influence patient’s QOL. However, results regarding the intensity of pain reported 2 days after surgery approached significance. The patients treated with the envelope flap (M = 3.86; sd = 2.43), on average, reported a more painful post-operative course than those who were treated with the intra-sulcular bayonet flap (M = 2.75; sd = 2.27). Furthermore, the bayonet flap (M = 1.43; sd = 0.69) was associated with higher levels of swelling after 14 days from surgery compared to the envelope flap (M = 1.14; sd = 0.36). Both aspects have been already studied by several studies and reviewed by Zhu et al. in 2020 [21]. No superiority of one or the other flaps in terms of pain was found in that review, except for Pell and Gregory Class 1 and 2 cases, in which significantly lower pain was found in the envelop flap than in the triangular flap. On the contrary, most authors found greater post-operative swelling with the triangular flap than with the envelope one [39,40,41]. Therefore, the flap choice does not seem to have relevant effects on the patient’s post-operative QOL, although delayed clinical healing impacts it [42].

Therefore, every effort should be made to achieve good healing and therefore improve the patient’s perception of outcomes.

### 4.5. Study Limitations

The data analysis of the present study often showed statistical non-significance, which is possibly related to the limited sample size and the poor numerical representativeness of some variables. Therefore, for a more in-depth understanding of the correlated variables, future research could examine a larger sample, so that each variable can be numerically more representative.

Furthermore, the possible variability in surgical techniques used for LTM extraction by different surgeons may have influenced the results. It would be interesting to evaluate the results of a similar study in which surgeries are carried out by a single expert surgeon, thus guaranteeing greater uniformity of the surgical procedure.

Lastly, edema could be assessed using more objective methods than simple clinical evaluation, although all assessments were consistently performed by the same expert surgeon.

## 5. Conclusions

The results of the present randomized clinical study showed that, after the extraction of fully impacted lower third molars, clinically relevant differences in wound healing were observed based on the type of access flap used. Specifically, patients treated with the envelope flap had larger dehiscence diameters than those treated with the intra-sulcular bayonet flap, exhibited more severe edema 2 days after surgery and showed lower clinical healing indices. Furthermore, the diameter of the dehiscence was negatively correlated with the clinical healing index.

As for the anatomical and operative variables, it was found that LTM root morphology and the depth of the residual bone cavity also have an influence on post-extraction healing, predisposing in some cases to the occurrence of dehiscence. Despite these differences, the flap-type does not seem to have relevant effects on the patient’s post-operative QOL.

## Figures and Tables

**Table 1:** Overall sample data by variables.

Variable		N (%)	± sd
Total patient sample		56 (100)	
Mean patient age (years)		23.7	4.48
Race	Caucasian	55 (98.21)	
	Asian	1 (1.79)	
Gender	Females	38 (67.86)	
	Males	18 (32.14)	
Mean pre-operative inter-incisal high (cm)			4.02
Mean FMPS (%)			29.70
Mean local plaque index score (%)			31
Pre-operative symtoms	Present	9 (16.07)	
	Absent	47 (83.93)	
Third molar site	Left	29 (51.79)	
	Right	27 (48.21)	
Radiographic third molar impaction type	Osteo-mucosal	48 (85.71)	
	Completely osseous	8 (14.29)	
Pell&Gregory Class	I	5 (8.93)	
	II	44 (78.57)	
	III	7 (12.50)	
Class of depth impaction	A	7 (12.50)	
	B	32 (57.14)	
	C1	15 (26.79)	
	C2	2 (3.57)	
Relationship with the second molar	Contiguity	44 (78.57)	
	Overlapping	8 (14.29)	
	None	4 (7.14)	
Third molar position	Mesio-angular	29 (51.79)	
	Horizontal	16 (28.57)	
	Disto-angular	2 (3.57)	
	Vertical	9 (16.07)	
Root number for each third molar	1	5 (8.93)	
	2	50 (89.29)	
	More than 2	1 (1.79)	
Root morphology	Separated, slightly divergent	38 (67.86)	
	Separated, highly divergent	2 (3.57)	
	Fused	15 (26.79)	
	With apical anomalies	1 (1.79)	
Mean pre-operative probing depth at the second molar distal surface (mm)			5
Mean amount of adherent gingiva, buccally to the second molar (mm)			2.5
Pre-operative soft tissue level in relation to the CEJ	Coronal	43 (76.79)	
	Apical	1 (1.79)	
	Normal	12 (21.43)	
Post-operative soft tissue level in relation to the CEJ	Coronal	43 (76.79)	
	Apical	1 (1.79)	
	Normal	12 (21.43)	
Mean maximum diameter of the final bone cavity (mm)			12 ± 2.61
Average maximum depth of the residual bone cavity in relation to the CEJ (mm)			9.72
Mean surgery duration without suturing time (seconds)			1520
Mean suturing duration (seconds)			481
Dehiscence at 2 days	Present	3 (5.36)	
	Absent	53 (94.64)	
Dehiscence at 7 days	Present	14 (25.00)	
	Absent	42 (75.00)	
Dehiscence at 14 days	Present	23 (41.07)	
	Absent	33 (58.93)	
Facial edema at 2 days	Mild	13 (23.21)	
	Moderate	31 (55.36)	
	Severe	12 (21.43)	
Facial edema at 7 days	Mild	42 (75.00)	
	Moderate	13 (23.21)	
	Severe	1 (1.79)	
Facial edema at 14 days	Absent	16 (28.57)	
	Mild	37 (66.07)	
	Moderate	3 (5.36)	
	Severe	0 (0.00)	
Mean dehiscence at 2 days (mm)		5	3.55
Mean dehiscence at 7 days (mm)		3.04	2.33
Mean dehiscence at 14 days (mm)		2.56	1.29
Mean healing index at 2 days		6	1.49
Mean healing index at 7 days		6.25	1.67
Mean healing index at 14 days		7.41	1.47

FMPS = Full Mouth Plaque Score; CEJ = cemento–enamel junction; sd = standard deviation.

**Table 2:** Independent samples *t*-test comparing the maximum diameter of dehiscence at 7 and 14 days after surgery.

	Envelope M (ds)	Bayonet M (ds)	*t*	*p*
14 days diameter	1.26 (1.80)	0.84 (1.18)	1.045	0.300
7 days diameter	1.00 (2.16)	0.54 (1.29)	0.976	0.333

M = Medium.

**Table 3:** General healing index ^§^ by flap type.

	General Healing Index ^§^		
Flap	Dehiscence	Average	Standard deviation
Envelope	Absent	1.7500	2.23607
	Present	0.1667	1.80067
	Total	1.0714	2.17611
Bayonet	Absent	1.9412	1.59963
	Present	1.1818	1.83402
	Total	1.6429	1.70434
Total	Absent	1.8485	1.90593
	Present	0.6522	1.84905
	Total	1.3571	1.95800

^§^ It was calculated based on the difference between the wound healing index detected at 14 and 2 days, respectively.

**Table 4:** Variance analysis for average values of the general healing index ^§^. Significant values are in bold.

	F	*p*
Flap	1.382	0.245
Dehiscence	**5.212**	**0.027**
Flap*Dehiscence	0.645	0.426

^§^ It was calculated based on the difference between the wound healing index detected at 14 and 2 days, respectively. * indicates an interaction between the two variables.

**Table 5:** Correlation analysis (Pearson’s r coefficient) between healing and dehiscence. Significant values are in bold.

	Maximum Diameter of Dehiscence at 7 Days		Maximum Diameter of Dehiscence at 14 Days	
Healing	R	p	r	p
2 days	−0.131	0.336	−0.065	0.635
7 days	−0.259	0.054	−0.218	0.106
14 days	−0.211	0.119	**−0.493**	**<0.001**
GHI	−0.059	0.664	**−0.325**	**0.015**

GHI = general healing index.

## Data Availability

The original contributions presented in this study are included in the article. Further inquiries can be directed to the corresponding author.
